# Morbus Castleman-assoziierter paraneoplastischer Pemphigus bei einer 16-jährigen Patientin

**DOI:** 10.1007/s00105-024-05328-5

**Published:** 2024-04-24

**Authors:** Sebastian T. Bender, Galina Balakirski, Walid Kteiche, Enno Schmidt, Silke C. Hofmann

**Affiliations:** 1 Zentrum für Dermatologie, Allergologie und Dermatochirurgie, Helios Universitätsklinikum Wuppertal, Universität Witten/Herdecke, Heusnerstr. 40, 42283 Wuppertal, Deutschland; 2 Klinik für Pneumologie, Ruhrlandklinik, Essen, Deutschland; 3 Lübeck Institut für Experimentelle Dermatologie, Universität Lübeck, Lübeck, Deutschland; 4 Klinik für Dermatologie, Allergologie und Venerologie, Universitätsklinikum Schleswig-Holstein, Campus Lübeck, Lübeck, Deutschland

**Keywords:** Paraneoplastisches autoimmunes Multiorgansyndrom, Bullöse Autoimmundermatose, Bronchiolitis obliterans, Autoantikörper, Plakine, Paraneoplastic autoimmune multiorgan syndrome, Bullous autoimmune disease, Bronchiolitis obliterans, Autoantibodies, Plakins

## Abstract

Der paraneoplastische Pemphigus ist eine seltene, lebensbedrohliche Autoimmunerkrankung, die klinisch durch meist ausgedehnte und therapierefraktäre Schleimhauterosionen und polymorphe Hautveränderungen charakterisiert ist. Wir berichten hier über eine 16-jährige Patientin mit isolierten oralen Erosionen, bei der zunächst ein Schleimhautpemphigoid vermutet wurde und es unter Therapie mit Prednisolon und Dapson zu einer deutlichen Befundbesserung kam. Allerdings entwickelte die Patientin einige Monate später eine massive respiratorische Insuffizienz infolge einer Bronchiolitis obliterans, sodass eine Lungentransplantation geplant wurde. Im Rahmen der vorbereitenden Diagnostik wurde ein unizentrischer, abdominell lokalisierter Morbus Castleman diagnostiziert, was schließlich zu der Diagnose eines paraneoplastischen Pemphigus mit Nachweis von Envoplakin-Autoantikörpern führte. Durch die Tumorresektion und anschließende Lungentransplantation konnte ein guter Allgemeinzustand wiederhergestellt werden bei anhaltender mukokutaner Remission.

## Abstract (en)

Paraneoplastic pemphigus is a rare, life-threatening autoimmune disease that is clinically characterized by mostly extensive and refractory mucosal erosions and polymorphous skin lesions. We report here on a 16-year-old girl with isolated oral erosions, in whom mucosal pemphigoid was initially suspected and after treatment with prednisolone and dapsone marked improvement was achieved. However, a few months later the patient developed massive respiratory insufficiency as a result of bronchiolitis obliterans, so that a lung transplant was planned. As part of the preparatory diagnostic workup, unicentric, abdominally localized Castlemanʼs disease was diagnosed, which ultimately led to the diagnosis of paraneoplastic pemphigus as evidenced by envoplakin autoantibodies. Tumor resection and subsequent lung transplantation achieved good results with sustained mucocutaneous remission.

Der paraneoplastische Pemphigus (PNP) ist eine seltene, schwer verlaufende bullöse Autoimmundermatose mit akut auftretenden, ausgeprägten und meist multilokulären mukokutanen Läsionen und möglicher Beteiligung weiterer Organsysteme [1, 2]. Diese obligate Paraneoplasie ist am häufigsten mit lymphoproliferativen oder hämatologischen Neoplasien, seltener mit soliden Tumoren assoziiert, die bei Diagnosestellung bereits bestehen oder später diagnostiziert werden [3]. Der klinische Befund ist charakterisiert durch ausgedehnte, chronische Erosionen v. a. an der Mundschleimhaut, aber auch an anderen Schleimhäuten sowie häufig auch polymorphen Hautveränderungen, die lichenoid oder kokardenartig sein können oder pralle Blasen aufweisen.

Die Autoantikörper binden an Harnblasenepithel in der indirekten Immunfluoreszenz und sind spezifisch gegen Plakine (v. a. Envoplakin), Alpha-2-Macroglobulin-1-Like (A2ML1), Desmogleine, Desmocolline oder BP180 gerichtet [2, 4]. Unbehandelt besitzt der PNP eine ungünstige Prognose. Die Identifikation und Therapie des zugrunde liegenden Tumors stellen die wichtigste Voraussetzung für eine erfolgreiche Behandlung der Erkrankung dar.

## Kasuistik

**Abb. 1:**
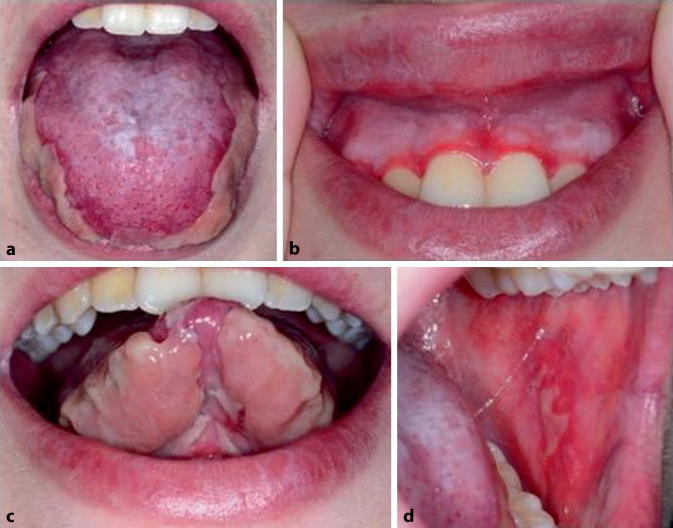
Mundschleimhautbefund bei Erstvorstellung mit ausgedehnten, fibrinbelegten Ulzerationen und Erosionen an der Zunge (**a, c**), der Gingiva (**b**) und der bukkalen Schleimhaut (**d**)

**Abb. 2:**
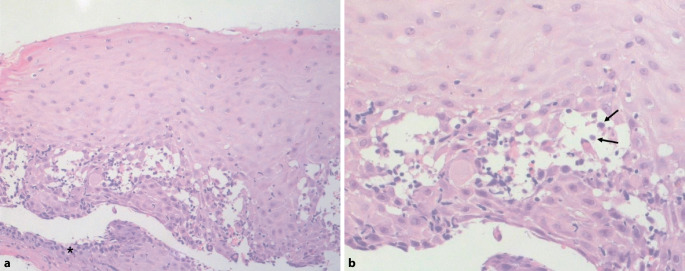
Histologie von einer Gingivabiopsie (Hämatoxylin-Eosin-Färbung). Zu erkennen sind Anteile des unverhornenden Plattenepithels der Mundschleimhaut und eine suprabasale intraepidermale Spaltbildung bei (mit *Stern* markierten) erhaltenen basalen Keratinozyten (**a**) (Vergr. 200:1). **b** Des Weiteren können abgerundete, akantholytische suprabasale Keratinozyten (mit *Pfeilen* markiert) festgestellt werden (Vergr. 400:1)

Eine 16-jährige, bisher gesunde Patientin stellte sich aufgrund neu aufgetretener, ausgedehnter, schmerzhafter Erosionen bukkal, gingival und an der Zunge vor (Abb. 1). Hautveränderungen am Körper bestanden nicht. Eine Biopsie der Gingiva zeigte eine suprabasale intraepidermale Spaltbildung mit akantholytischen suprabasalen Keratinozyten (Abb. 2a, b). Die direkte Immunfluoreszenz (DIF) von bukkal sowie die indirekte Immunfluoreszenz (IIF) auf Affenösophagus waren negativ. In der IIF auf Spalthaut wurden IgG-Ablagerungen am Blasendach nachgewiesen bei fehlendem Nachweis von spezifischen Autoantikörpern gegen BP180, LAD‑1, BP230, Kollagen VII, Laminin 332 und Desmoglein 3, sodass am ehesten von einem oralen Schleimhautpemphigoid ausgegangen wurde.

**Abb. 3:**
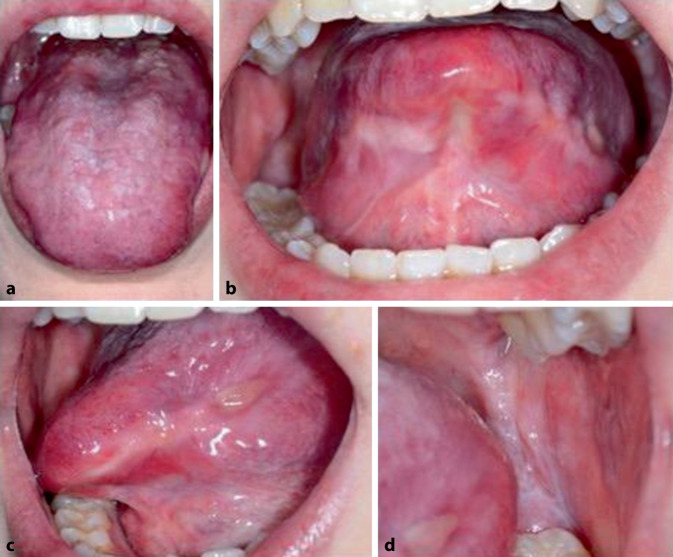
Verlaufskontrolle nach 3 Monaten Systemtherapie mit Prednisolon (zu diesem Zeitpunkt noch 17,5 mg/Tag) und Dapson. Es zeigte sich eine deutliche Befundbesserung mit deutlichem Rückgang der Läsionen an der Zunge (**a–c**) und der bukkalen Schleimhaut (**d**)

Unter einer Therapie mit initial 75 mg/Tag Prednisolon und 75 mg/Tag Dapson besserte sich der Schleimhautbefund (Abb. 3). Etwa 5 Monate nach Erstvorstellung klagte die Patientin über eine progrediente Belastungsdyspnoe, lehnte aber aufgrund der vorherrschenden COVID-19-Pandemie eine Vorstellung in der Klinik ab. Bei stabilem Schleimhautbefund wurde empfohlen, einen Pneumologen aufzusuchen und Dapson abzusetzen aus Sorge vor einer eosinophilen Pneumonie als Ursache der Dyspnoe [5].

**Abb. 4:**
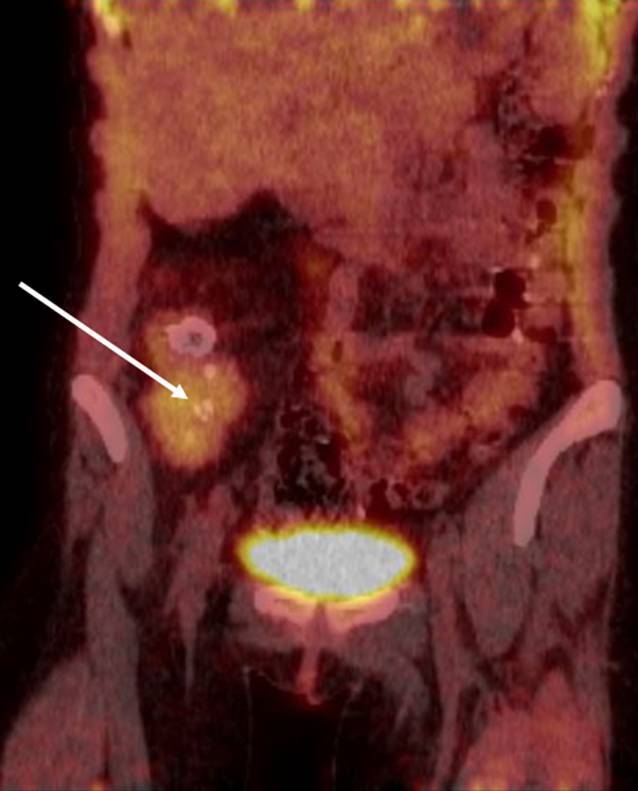
PET-CT-Aufnahme mit Nachweis eines stoffwechselaktiven Tumors im Unterbauch rechts (mit *Pfeil* markiert)

Vier Monate später wurde die Patientin wegen progredienter respiratorischer Insuffizienz notfallmäßig in eine pneumologische Fachklinik eingewiesen. Zu diesem Zeitpunkt bestanden keine Schleimhautläsionen mehr, und auch der Hautbefund war blande. Bei der inzwischen 17-jährigen Patientin wurde bei deutlich reduzierter Einsekundenkapazität (FEV_1_) auf 19 % des Sollwertes mittels Computertomographie die Diagnose einer ausgeprägten Bronchiolitis obliterans gestellt, sodass eine Lungentransplantation erwogen werden musste. Zur Vorbereitung erfolgte eine umfangreiche bildgebende Diagnostik, wobei ein Tumor im Unterbauch diagnostiziert (Abb. 4) und exstirpiert wurde. In Zusammenschau der Bildgebung und der Histopathologie ergab sich die Diagnose eines unizentrischen Morbus Castleman. Bei fehlender Besserung der Lungenfunktion unter einer immunmodulatorischen Therapie mit Tocilizumab erfolgte im Verlauf eine bilaterale sequenzielle Lungentransplantation.

**Abb. 5:**
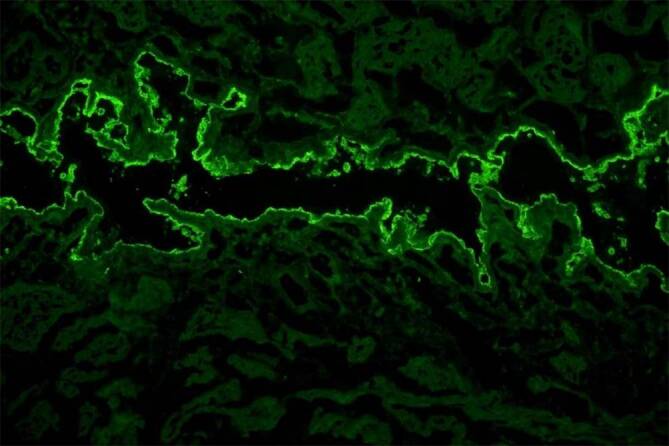
Indirekte Immunfluoreszenz auf Rattenblase: Zirkulierende IgG-Autoantikörper binden an das Plakin-reiche Urothel (Vergr. 200:1)

**Abb. 6:**
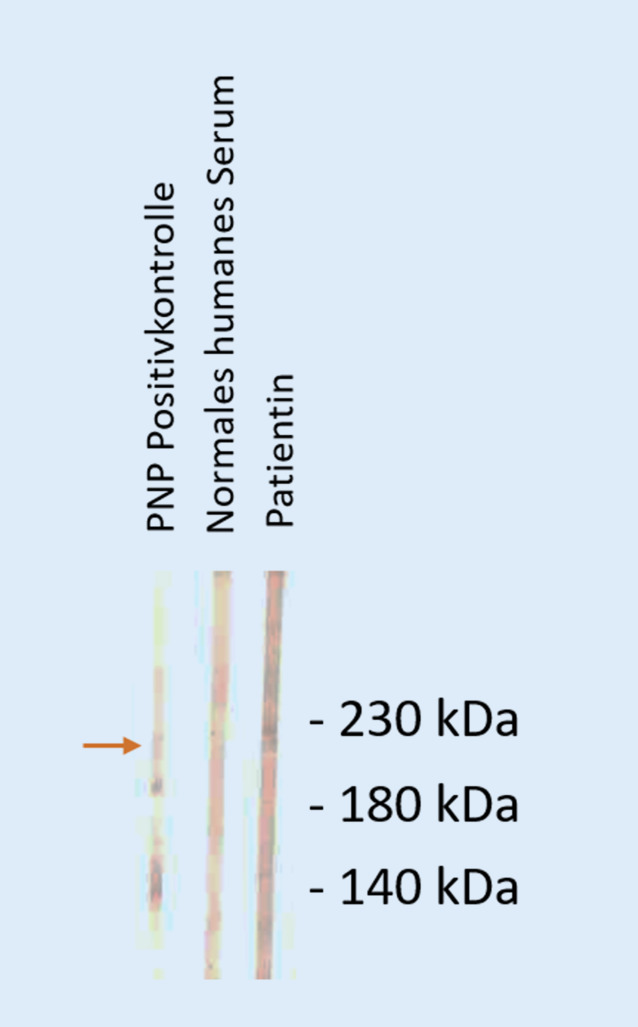
Im Immunoblot mit Keratinozytenextrakt reagiert das Serum der Patientin mit dem 210 kDA großen Envoplakin

Die Diagnose einer Bronchiolitis obliterans bei Morbus Castleman veranlasste uns zur Ausweitung der serologischen Diagnostik. Sowohl in dem initial kryoasservierten Serum wie auch in einem aktuellen Serum der Patientin zeigte sich die IIF auf Rattenblase positiv (Abb. 5). Im Immunoblot mit Extrakt kultivierter humaner Keratinozyten und ELISA mit rekombinantem Envoplakin (Euroimmun, Lübeck) ließen sich zirkulierende Autoantikörper nachweisen (Abb. 6). Demzufolge wurde die Diagnose revidiert und ein paraneoplastischer Pemphigus mit Bronchiolitis obliterans im Sinne eines paraneoplastischen autoimmunen Multiorgansyndroms (PAMS) diagnostiziert.

Zwei Jahre nach der Lungentransplantation befindet sich die Patienten unter Immunsuppression mit Tacrolimus und Mycophenolat-Mofetil in einem stabilen Allgemeinzustand bei guter Transplantatfunktion und fehlendem Anhalt für Haut- oder Schleimhautläsionen.

## Diskussion

Der paraneoplastische Pemphigus (PNP) wurde erstmalig durch den US-amerikanischen Dermatologen Grant James Anhalt im Jahr 1990 beschrieben [1]. Es handelt sich um eine sehr seltene polymorphe mukokutane Erkrankung, die auf autoimmunvermittelten Prozessen mit Bildung von IgG-Autoantikörpern gegen Zell-Zell-Adhäsionsproteine der suprabasalen Keratinozyten beruht. Vermutlich wird der PNP durch die humorale und zelluläre Immunantwort des Körpers auf Tumorzellen ausgelöst [4].

Im Gegensatz zum Pemphigus vulgaris, der sich mit typischen Erosionen der Haut und Schleimhaut präsentiert, ist das klinische Bild des PNP sehr heterogen. Charakteristisch ist eine schwere, erosive Stomatitis mit einer hämorrhagischen Cheilitis. Häufig findet sich zudem eine Beteiligung der Zunge wie bei unserer Patientin. An Stamm und Extremitäten kommen Pemphigus-artige Erosionen ebenso vor wie Läsionen, die an einen Lichen ruber planus, ein Erythema exsudativum multiforme oder ein Stevens-Johnson-Syndrom erinnern. Ebenso wurden zahlreiche mögliche Autoantigene beschrieben, wobei insbesondere Autoantikörper gegen Proteine der Plakin-Familie (Envoplakin, Desmoplakin, Periplakin und Epiplakin) sowie gegen A2LM1 spezifisch für PNP sind [2]. Da diese Ankerproteine in unterschiedlichem Ausmaß in allen Epithelien vorkommen, erklärt sich die Manifestation der Erkrankung an verschiedenen Organen und somit die Entstehung eines paraneoplastischen autoimmunen Multiorgansyndroms (PAMS) [2–4].

Wie auch in dem vorliegenden Fall gehen in etwa einem Drittel der Fälle (Schleim‑)Hautläsionen einer Neoplasie bzw. deren Diagnose voraus [6]. Die histologischen Veränderungen können in Abhängigkeit von der klinischen Manifestation variieren. Liegt eine ausgeprägte Stomatitis vor, so zeigen viele Biopsien eine unspezifische Entzündung oder Ulzerationen. Von Vorteil sind periläsional entnommene Biopsien, welche oft eine suprabasale Akantholyse aufweisen. Im Gegensatz zu anderen Formen des Pemphigus kommen bei PNP häufig entzündliche Infiltrate im Bereich der frühen Läsionen vor. Dabei kann das histologische Bild mit einzelnen Keratinozytennekrosen und lymphozytärem Infiltrat in der Epidermis in Biopsien von nichtbullösen Läsionen an ein Erythema exsudativum multiforme oder eine Graft-versus-Host-Krankheit denken lassen. Andererseits können Bereiche mit einer Interface-Dermatitis und vakuolären Degeneration der basalen Keratinozyten den kutanen Lupus erythematodes imitieren [7].

In der DIF kann nicht nur eine Ablagerung von IgG und Komplement in den Interzellularräumen der suprabasalen Keratinozyten, sondern auch entlang der Basalmembranzone vorkommen. Allerdings kann die DIF bei PNP in bis zu 50 % der Fälle negativ sein [7, 8]. Somit wurde bei unserer Patientin aufgrund des nur umschriebenen oralen Schleimhautbefundes, des guten Allgemeinbefindens, der bandförmigen IgG-Ablagerung am Blasendach auf Spalthaut in der indirekten Immunfluoreszenz und des negativen Desmoglein-ELISA trotz der histologisch nachgewiesenen suprabasalen Spaltbildung mit Vorhandensein einzelner akantholytischer Keratinozyten die Diagnose eines oralen Schleimhautpemphigoids favorisiert. Erst die Kombination der im Verlauf hinzugetretenen Diagnosen einer Bronchiolitis obliterans und eines bis zum Diagnosezeitpunkt asymptomatischen Morbus Castleman führten zur Ergänzung der serologischen Diagnostik im Hinblick auf einen PNP. Neben dem Nachweis der Autoantikörper gegen Envoplakin oder A2LM1 stellt die positive indirekte Immunfluoreszenz auf Rattenblase einen hochspezifischen Parameter zur Unterscheidung des PNP von einem Pemphigus vulgaris dar [9, 10].

Als häufig mit PNP assoziierte Neoplasien werden Non-Hodgkin-Lymphome, chronisch lymphatische Leukämie, maligne Thymome, Sarkome und Morbus Castleman angesehen [11–13]. Bei dem Letzteren handelt es sich um eine seltene lymphoproliferative Erkrankung, die im Jahr 1954 zum ersten Mal durch den amerikanischen Pathologen Benjamin Castleman beschrieben wurde. Sie besitzt charakteristische histopathologische Merkmale, ist aber durch sehr variable Verlaufsformen charakterisiert. So ist die unizentrische Form der Erkrankung mit lokalisiertem Lymphknotenbefall oft ein Zufallsbefund. In über 90 % der Fälle ist die operative Sanierung bei unizentrischem Morbus Castleman kurativ und führt insofern auch bei assoziiertem PNP meist zu einer kompletten Remission mit Rückgang der Autoantikörper. Dagegen besitzt die seltenere multizentrische Form des Morbus Castleman mit mindestens 2 betroffenen Regionen eine deutlich schlechtere Prognose verbunden mit einer hohen Mortalität [12].

Ein Teil der PNP-Patienten, in Europa ca. 25 %, entwickelt eine Lungenbeteiligung in Form einer Bronchiolitis obliterans oder organisierenden Pneumonie [14]. Allerdings liegt dieser Anteil bei Patienten mit PNP und unizentrischem Morbus Castleman deutlich höher. In der vor Kurzem beschriebenen großen europäischen Kohorte hatten 57 % der Patienten diese Komplikation entwickelt [15]. Somit muss bei PNP-Patienten mit assoziiertem Morbus Castleman von einem höheren Risiko für eine Lungenbeteiligung ausgegangen werden, und die Patienten sollten hierüber aufgeklärt werden.

Da bei unserer Patientin auch aufgrund des sehr guten Ansprechens auf die eingeleitete Therapie ein PNP initial nicht in Betracht gezogen wurde, ist eine derartige Aufklärung leider nicht erfolgt, was sicherlich auch die verspätete Diagnose einer Bronchiolitis obliterans bei länger anhaltender Dyspnoe begünstigt hat. Wie auch in dem hier beschriebenen Fall kommt es meist im Rahmen der Lungenbeteiligung bei PNP trotz Behandlung der Neoplasie zu einer irreversiblen und progredienten Destruktion der Bronchien mit konsekutiver Lungeninsuffizienz, die grundsätzlich mit einer schlechten Prognose assoziiert ist [16–18]. Ohne erfolgreiche Therapie des assoziierten Malignoms ist die Mortalität bei PNP infolge eines Lungenversagens daher sehr hoch [19]. Die einzig wirksame Therapie einer Bronchiolitis obliterans stellt somit die bilaterale Lungentransplantation dar [20], die aber nur bei erfolgreich behandeltem Malignom in Betracht kommt.

Eine optimale Therapie der Tumorerkrankung ist somit für das Überleben der Patienten entscheidend. So kann beispielsweise bei chronisch lymphatischer Leukämie mit dem Tyrosinkinaseinhibitor Ibrutinib behandelt und eine Befundstabilisierung der Bronchiolitis obliterans mit deutlicher Verlängerung des Überlebens erreicht werden [21].

Glücklicherweise ermöglichte die Diagnose eines vollständig exstirpierten unizentrischen Morbus Castleman bei unserer Patientin die erfolgreiche Lungentransplantation. Insgesamt scheinen PNP-Patienten mit Morbus Castleman eine günstigere Prognose zu haben verglichen mit anderen zugrunde liegenden Neoplasien. Dagegen werden Erythema-multiforme-artige Hautveränderungen mit einer schlechteren Prognose assoziiert [14].

Dieser Fall verdeutlicht die diagnostischen Schwierigkeiten bei dem seltenen und klinisch sehr heterogenen Krankheitsbild PNP, die Notwendigkeit einer klinisch-histologischen Korrelation bei oft unspezifischer Histologie sowie die hohe diagnostische Relevanz der indirekten Immunfluoreszenz auf Rattenblase und des Envoplakin ELISA. Wie in der kürzlich publizierten Leitlinie zum Management des PNP beschrieben [2], sollten diese diagnostischen Methoden bei atypischer Klinik einer blasenbildenden Autoimmunerkrankung oder bei kombinierter Antikörper- oder Komplementbindung an epidermale Keratinozyten sowie Basalmembranzone in der direkten Immunfluoreszenz stets durchgeführt werden, um einen PNP auszuschließen. Für eine günstige Prognose ist, wie in dem hier beschriebenen Fall, eine interdisziplinäre Behandlung essenziell.
